# Protocol Adherence for Severe Sepsis and Septic Shock Management in the Emergency Department; a Clinical Audit

**Published:** 2017-01-09

**Authors:** Mostafa Alavi-Moghaddam, Ali Anvari, Reaza Soltani Delgosha, Hamid Kariman

**Affiliations:** 1 Emergency Department, Imam Hossein Hospital, Shahid Beheshti University of Medical Sciences, Tehran, Iran.; 2 Emergency Department, Shohadaye Pakdasht Hospital, Shahid Beheshti University of Medical Sciences, Tehran, Iran.

**Keywords:** Sepsis, shock, septic, disease management, guideline adherence, clinical audit

## Abstract

**Introduction:**

Although significant development in the field of medicine is achieved, sepsis is still a major issue threatening humans’ lives. This study was aimed to audit the management of severe sepsis and septic shock patients in emergency department (ED) according to the present standard guidelines.

**Method:**

This is a prospective audit on approaching adult septic patients who were admitted to ED. The audit checklist was created based on the protocols of Surviving Sepsis Campaign and British Royal College recommendations. The mean knowledge score and the compliance rate of studied measures regarding standard protocols were calculated using SPSS version 21.

**Results:**

30 emergency medicine residents were audited (63.3% male). The mean knowledge score of studied residents regarding standard guidelines were 5.07 ± 1.78 (IQR = 2) in pre education and 8.17 ± 1.31 (IQR = 85) in post education phase (p < 0.001). There was excellent compliance with standard in 4 (22%) studied measures, good in 2 (11%), fair in 1 (6%), weak in 2 (11%), and poor in 9 (50%). 64% of poor compliance measures correlated to therapeutic factors. After training, score of 5 measures including checking vital signs in < 20 minute, central vein pressure measurement in < 1 hour, blood culture request, administration of vasopressor agents, and high flow O_2_ therapy were improved clinically, but not statistically.

**Conclusion:**

The protocol adherence in management of severe sepsis and septic shock for urine output measurement, central venous pressure monitoring, administration of inotrope agents, blood transfusion, intravenous antibiotic and hydration therapy, and high flow O_2 _delivery were disappointingly low. It seems training workshops and implementation of Clinical audit can improve residents’ adherence to current standard guidelines regarding severe sepsis and septic shock.

## Introduction

Sepsis is a critical condition, which is characterized by immune system response to bacterial infections that can lead to acute organ failure (1-4). Despite significant developments in the field of medicine, sepsis is still a major issue threatening humans’ lives (5). The increasing incidence rate of severe sepsis and septic shock during the past three decades has led to sepsis becoming the second main cause of death among shock patients. The mortality rate among septic patients strongly correlates with organ dysfunction (6). Based on previous studies, the mortality rate of severe sepsis and sepsis shock were 25-30% and 40-70%, respectively (7, 8). Controlling the inflammation processes can prevent sepsis from turning into septic shock and damage to vital organs, therefore, decrease the mortality and morbidity of these patients (9). According to this theory, Surviving Sepsis Campaign recommended a guideline with the aim of diagnosis and treatment of septic patients to improve the prognosis (10). Institute for Healthcare Improvement also recommend protocols for resuscitation of severe sepsis and septic shock patients in the first four hours of diagnosis. However, still many defects exist in approaching and managing these patients (11-14)(19-22). This study was aimed to audit the management of severe sepsis and septic shock patients in emergency department (ED) according to the present standard guidelines before and after the training workshop

## Methods

***Study design***

This is a prospective audit on approaching adult septic patients who were admitted to the ED of Imam Hossein educational Hospital, Tehran, Iran, during October 2010 to May 2011. The study protocol was approved by ethics committee of Shahid Beheshti University of Medical Sciences. Researchers adhered to all Helsinki recommendations and confidentiality of patient profiles during the study period.

***Data Gathering***

Data gathering was performed using a predesigned standard checklist and convenience sampling method. The audit checklist was created based on the protocols of Surviving Sepsis Campaign and British Royal Collage recommendations (14-16). Checklist items were categorized into two groups of diagnostic and treatment measures. These measures consisted of checking vital signs within 20 minutes of admission (blood pressure, pulse rate, respiratory rate, temperature, oxygen saturation), blood sugar, arterial blood gas (ABG) parameters, urine output; blood culture request; inserting central venous line and checking central venous pressure in the first 2 hours of admission; ordering and administration of high flow oxygen; fluid resuscitation with crystalloid; antibiotic therapy; administration of vasopressor and positive inotrope agents; blood transfusion; and orotracheal intubation.

Minimum ideal compliance rate for each measures according to the local condition were defined as follows: checking vital sign and ABG for 95% of patients; administration of high flow oxygen for 95%; administration of intravenous fluid for 75% in the first hour of admission, 90% in the second hour, and 100 before leaving ED; initiation of intravenous antibiotic for 50% in the first hour, 90% in the second hour, and 100% before leaving ED; and checking urine output for 90% before leaving ED.

***Audit phases***

In the first phase of study (about 3 months), management of septic patients by emergency medicine residents was evaluated using the mentioned checklist and the time from ED presentation to reaching a diagnosis was recorded. A trained emergency medicine resident was responsible for real time checking and recording of required items for each patients. Then, the faults and shortcomings of management were extracted and a training workshop was held for all in charge emergency medicine residents. In the second phase (1 month after finishing education) performance of the same residents in management of septic shock and severe sepsis was reevaluated using the same checklist (about 3 months).

***Statistical analysis***

Data were analyzed using SPSS version 21. Variables were presented as frequency and percentage, inter quartile range (IQR), and mean ± standard deviation. The compliance rates were categorized into five groups based on Likert scale: ≥ 90% as excellent (score 5), 80-90% good (score 4), 70-80% fair (score 3), 60-70% weak (score 2) and < 60% poor (score 1). Comparisons were made using student t test, Wilcoxon, and chi square tests. P < 0.05 was considered as statistically significant. 

## Results

30 emergency medicine residents were audited regarding management of severe sepsis and septic shock. The mean knowledge score of studied residents regarding standard guidelines were 5.07 ± 1.78 (IQR = 2) in pre education and 8.17 ± 1.31 (IQR = 2) in post education phase (p < 0.001). The median time from admission to diagnosis were 55 and 15 minutes in pre and post training phases, respectively (p < 0.001). There were excellent compliance with standard in 4 (22%) studied measures, good in 2 (11%), fair in 1 (6%), weak in 2 (11%), and poor in 9 (50%). 64% of poor compliance measures correlated to therapeutic factors. Table 1 compares compliance rate of different studied measures with standard guidelines between pre and post training periods.

After training, score of 5 measures including checking vital signs in < 20 minutes, central vein pressure measurement in < 1 hour, blood culture request, administration of vasopressor agents, and high flow O_2_ therapy were improved clinically, but not statistically.

**Table 1:** Comparison of compliance rate of different studied measures with standard guidelines between pre and post education periods

Studied measures	Pre Education		Post Education		P value
	% (n)	Rate*	% (n)	Rate	
Vital signs (<20 minute)	86.7 (26)	Good	90 (27)	Excellent	0.999
O_2 _saturation ( <20 minute)	86.7 (26)	Good	90 (27)	Excellent	1.000
Blood sugar	93.3 (28)	Excellent	96.7 (29)	Excellent	1.000
Urine output	20.0 (6)	Poor	33.3 (10)	Poor	0.382
Arterial blood gas	100 (30)	Excellent	100 (30)	Excellent	-
Blood culture	73.3 (22)	Fair	86.7 (26)	Good	0.333
Central venous pressure	60.0 (3)	Weak	71.4 (4)	Fair	1.000
Saturation central vein	0 (0)	Poor	37.5 (2)	Poor	0.209
Central venous line (<60 minute)	7.1 (1)	Poor	13.3(2)	Poor	1.000
Central venous line (>60 minute)	28.6 (4)	Poor	26.7 (4)	Poor	1.000
Bolus fluid therapy	28.6 (8)	Poor	40 (13)	Poor	0.700
Antibiotic therapy	60 (18)	Weak	60 (18)	Weak	1.000
Vasopressor administration	40 (2)	Poor	100 (6)	Excellent	0.182
Blood transfusion	0 (0)	Poor	50 (3)	Poor	0.229
Inotrope administration	0 (0)	Poor	50 (3)	Poor	0.497
Rapid sequence intubation	100 (7)	Excellent	100 (9)	Excellent	-
O_2 _therapy	96.7 (29)	Excellent	100 (30)	Excellent	1.000
High flow O_2 _therapy	10 (3)	Poor	40 (12)	Poor	0.015

* Based on Likert scale: ≥ 90% as excellent, 80-90% good, 70-80% fair, 60-70% weak and < 60% poor.

## Discussion

Based on the findings of the present study, there was fair (70-80%) to poor (<60%) compliance with standard protocol regarding 64% of studied measures in management of severe sepsis and septic shock. It reduced to 55% after training workshops. The protocol adherence for urine output measurement, central venous pressure monitoring, administration of inotrope agents, blood transfusion, intravenous antibiotic and hydration therapy, and high flow O_2 _delivery were disappointingly low in both pre and post training periods. The mean time from arrival to ED and reaching diagnosis was significantly decreased after training. 

The study of Miller et al. showed that by performing the protocols accurately, the rate of death decreased (12). Catenacci et al. reported that evaluating the severe sepsis patients according to protocols caused 16 percent decrements in the rate of mortality (17). 

Administrating high flow oxygen was significantly increased from 10% to 40% of patients after training, but it was in poor compliance with sepsis treatment protocols. Due to normal oxygen saturation in a large proportion of septic patients, emergency residents did not order high flow oxygen for them, wrongly. 

Kumar et al. evaluated 2731 patients with severe sepsis and septic shock in the United states and Canada, and found out that administration of antibiotics in the first hour of admission can improve survival rate by 79.9%, whereas, as they observed, each one-hour delay in antibiotic administration can increase mortality rate by 7.9% (18). In addition, Leibovici et al. demonstrated that antibiotic therapy in the first hour of presenting to ED can decrease mortality, significantly (19). 

In this study, intravenous fluid was properly administered for only 28.6% and 40% of patients before and after training. Administration of vasopressors agent was significantly increased after training workshops and reached excellent level of compliance with protocol. An audit that evaluated protocol adherence regarding fluid therapy in management of septic children showed that in 62% of shocked cases, guideline was not followed (20).

Since severe sepsis and septic shock patients are usually critically ill and have a high mortality rate, their management in the crowded ED is usually accompanied by hazards. Lack of fixed nursing and medical personnel for accurate and continuous monitoring of these patients, especially in the initial hours of arrival, worsens the situation. Under this condition, inevitably, all or part of the necessary diagnostic or therapeutic measures will be missed. As can be seen, even holding workshops in this regard could not significantly improve the situation. In other words, the main problem might not be proper knowledge, and the key to solve this problem might be found in the practice phase. Maybe more rapid disposition of these patients to intensive care unit or increasing the number of personnel and treatment equipment for these patients could be helpful. Preparing standard and logical checklists and requiring in-charge physicians to adhere to these protocols may be of help in this regard.

***Limitations***

It would have been better if by increasing sample size we could carry out sub-group analyses based on residency year, sex, type of shift, etc.

## Conclusion:

Based on the finding of the present study, there were fair (70-80%) to poor (<60%) compliance with standard protocol regarding 64% and 55% of studied measures in management of severe sepsis and septic shock in pre and post training workshops, respectively. The protocol adherence for urine output measurement, central venous pressure monitoring, administration of inotrope agents, blood transfusion, intravenous antibiotic and hydration therapy, and high flow O_2 _delivery were disappointingly low in both pre and post training period. It seems training workshops and implementation of clinical audit can improve residents’ adherence to current standard guidelines regarding severe sepsis and septic shock. 

## References

[1] Bone RC, Balk RA, Cerra FB (1992). Definitions for sepsis and organ failure and guidelines for the use of innovative therapies in sepsis. Chest.

[2] Tannehill D (2013). Treating Severe Sepsis & Septic Shock in 2012. Journal of Blood Disorders & Transfusion.

[3] Morrell MR, Micek ST, Kollef MH (2009). The management of severe sepsis and septic shock. Infectious disease clinics of North America.

[4] Stearns-Kurosawa DJ, Osuchowski MF, Valentine C, Kurosawa S, Remick DG (2011). The pathogenesis of sepsis. Annual review of pathology.

[5] Angus DC, Linde-Zwirble WT, Lidicker J, Clermont G, Carcillo J, Pinsky MR (2001). Epidemiology of severe sepsis in the United States: analysis of incidence, outcome, and associated costs of care. Critical Care Medicine-Baltimore-.

[6] Martin GS, Mannino DM, Eaton S, Moss M (2003). The epidemiology of sepsis in the United States from 1979 through 2000. New England Journal of Medicine.

[7] Bennett JE, Dolin R, Blaser MJ (2014). Principles and practice of infectious diseases.

[8] Fauci AS Harrison's principles of internal medicine.

[9] Russell JA (2006). Management of sepsis. New England Journal of Medicine.

[10] Vincent J-L, Moreno R, Takala J (1996). The SOFA (Sepsis-related Organ Failure Assessment) score to describe organ dysfunction/failure. Intensive care medicine.

[11] Ferrer R, Artigas A, Levy MM (2008). Improvement in process of care and outcome after a multicenter severe sepsis educational program in Spain. Jama.

[12] Miller III RR, Dong L, Nelson NC (2013). Multicenter implementation of a severe sepsis and septic shock treatment bundle. American journal of respiratory and critical care medicine.

[13] Robson W, Beavis S, Spittle N (2007). An audit of ward nurses’ knowledge of sepsis. Nursing in Critical Care.

[14] Dellinger RP, Levy MM, Rhodes A (2013). Surviving Sepsis Campaign: international guidelines for management of severe sepsis and septic shock, 2012. Intensive care medicine.

[15] Dellinger R, Carlet J, Masur H (2004). Surviving Sepsis Campaign Management Guidelines Committee: Surviving Sepsis Campaign guidelines for management of severe sepsis and septic shock. Crit Care Med.

[16] Dellinger RP, Levy MM, Carlet JM (2008). Surviving Sepsis Campaign: international guidelines for management of severe sepsis and septic shock: 2008. Intensive care medicine.

[17] Catenacci MH, King K (2008). Severe sepsis and septic shock: improving outcomes in the emergency department. Emergency medicine clinics of North America.

[18] Kumar A, Roberts D, Wood KE (2006). Duration of hypotension before initiation of effective antimicrobial therapy is the critical determinant of survival in human septic shock. Critical care medicine.

[19] Leibovici L, Shraga I, Drucker M, Konigsberger H, Samra Z, Pitlik S (1998). The benefit of appropriate empirical antibiotic treatment in patients with bloodstream infection. JOURNAL OF INTERNAL MEDICINE-OXFORD-.

[20] Inwald DP, Tasker RC, Peters MJ, Nadel S (2009). Emergency management of children with severe sepsis in the United Kingdom: the results of the Paediatric Intensive Care Society sepsis audit. Archives of disease in childhood.

